# Anti-c-fms Antibody Prevents Osteoclast Formation and Bone Resorption in Co-Culture of Osteoblasts and Osteoclast Precursors In Vitro and in Ovariectomized Mice

**DOI:** 10.3390/ijms21176120

**Published:** 2020-08-25

**Authors:** Yasuhiko Nara, Hideki Kitaura, Saika Ogawa, Wei-Ren Shen, Jiawei Qi, Fumitoshi Ohori, Takahiro Noguchi, Aseel Marahleh, Adya Pramusita, Ria Kinjo, Itaru Mizoguchi

**Affiliations:** Division of Orthodontics and Dentofacial Orthopedics, Tohoku University Graduate School of Dentistry, Aoba-ku, Sendai, Miyagi 980–8575, Japan; yasuhiko.nara.q6@dc.tohoku.ac.jp (Y.N.); saika.ogawa.a4@tohoku.ac.jp (S.O.); shen.wei.ren.t5@dc.tohoku.ac.jp (W.-R.S.); qi.jiawei.p8@dc.tohoku.ac.jp (J.Q.); fumitoshi.ohori.t3@dc.tohoku.ac.jp (F.O.); takahiro.noguchi.r4@dc.tohoku.ac.jp (T.N.); marahleh.aseel.mahmoud.t6@dc.tohoku.ac.jp (A.M.); adya.pramusita.q6@dc.tohoku.ac.jp (A.P.); ria.kinjou.p5@dc.tohoku.ac.jp (R.K.); mizo@tohoku.ac.jp (I.M.)

**Keywords:** osteoclast, OVX, anti-c-fms antibody, M-CSF

## Abstract

Osteoporosis morphology is characterized by bone resorption and decreases in micro-architecture parameters. Anti-osteoporosis therapy targets osteoclasts because bone resorption is a unique function of osteoclasts. Anti-c-fms antibodies against the receptor for macrophage colony-stimulating factor (M-CSF) inhibit osteoclast formation and bone resorption in vitro and in vivo. However, the effect of anti-c-fms antibodies on bone resorption in ovariectomized (OVX) mice is unknown. In this study, we evaluated the effect of anti-c-fms antibodies on osteoclast formation and bone resorption in osteoblast–osteoclast precursor co-culture in vitro and in OVX mice. Osteoblast and osteoclast precursor co-cultures treated with anti-c-fms antibodies showed significantly inhibited osteoclast formation, while cultures without anti-c-fms antibody treatment showed osteoclast formation. However, anti-c-fms antibodies did not change the receptor activator of nuclear factor kappa-B ligand (RANKL) or osteoprotegrin (OPG) expression during osteoblast and osteoclast differentiation in vitro. These results indicate that anti-c-fms antibodies directly affected osteoclast formation from osteoclast precursors in co-culture. OVX mice were treated with intraperitoneal injections of anti-c-fms antibody. The trabecular bone structure of the femur was assessed by micro-computer tomography. The anti-c-fms antibody inhibited osteoclast formation and bone loss compared with PBS-treated OVX mice. These results indicate potential for the therapeutic application of anti-c-fms antibodies for postmenopausal osteoporosis.

## 1. Introduction

Osteoporosis is a bone degradation disease, predominantly of postmenopausal women, that occurs worldwide [1]. Therefore, osteoporosis is a global health problem and an important area of research in many countries around the world [2]. Postmenopausal osteoporosis is a metabolic disease that causes decreasing bone mass and bone mineral density, and increased skeletal fragility and fracture risk as a result of hormone changes with age that increase osteoclast bone resorption [3,4,5]. Current treatments for osteoporosis include calcium, vitamin D, bisphosphonates, teriparatide, parathyroid hormone, denosumab and selective estrogen receptor modulators [6]. However, these treatments often have side effects and their therapeutic effects are less than ideal [7]. Therefore, we aim to find new treatments and to develop drugs for the prevention and treatment of postmenopausal osteoporosis.

Osteoclasts derived from bone marrow cells are associated with bone resorption and bone remodeling. Essential factors of osteoclast formation include the cytokines, macrophage colony-stimulating factor (M-CSF) and receptor activator of nuclear factor kappa-B ligand (RANKL) [8]. Tumor necrosis factor-α (TNF-α) also induces osteoclast formation in vitro [9,10,11] and in vivo [12,13]. Conversely, osteoprotegerin (OPG), which is a RANKL decoy receptor, prevents osteoclast formation [14]. Antibodies against the M-CSF receptor, c-fms, (anti-c-fms antibody) inhibit RANKL-induced osteoclast formation and TNF-α-induced osteoclast formation in vitro. In vivo, administration of an anti-c-fms antibody also inhibits bone erosion induced by TNF-α administration. Furthermore, anti-c-fms antibody administration inhibits osteoclast formation and bone erosion in experimental inflammatory arthritis [13]. Anti-c-fms antibodies also inhibit lipopolysaccharide (LPS)-induced osteoclast formation and bone resorption in mouse calvariae [15] and osteoclast formation in experimental periodontal diseases [16]. Anti-c-fms antibodies inhibit osteoclast formation and bone resorption on the pressure side of teeth during orthodontic tooth movement and may therefore be able to control orthodontic tooth movement [17]. Moreover, anti-c-fms antibodies significantly inhibit odontoclast formation and pathological root resorption in orthodontic tooth movement [18] and inhibit orthodontic relapse after orthodontic tooth movement [19]. M-CSF, a pleiotropic cytokine, is not only important for osteoclast differentiation but also proliferation, survival, regulation of key functions of monocytes and macrophages and their differentiation into mature phagocytes. M-CSF also plays an important role for phagocyte-mediated host defense such as bacterial infection and increased tumor cell cytotoxicity [20,21].

RANKL is synthesized as a membrane-bound molecule. Membrane-bound RANKL is cleaved into soluble RANKL by proteases [22,23,24]. Deficiency of soluble RANKL does not affect bone loss in an ovariectomy (OVX) model, indicating a role for membrane-bound RANKL via cell–cell contact in osteoporosis [25]. In in vitro co-culture of osteoblasts and osteoclast precursors, osteoclast formation requires signaling via soluble RANKL-mediated interactions or via membrane-bound RANKL through cell–cell contact. Osteoclast formation induced by soluble RANKL is inhibited by anti-c-fms antibodies [26]. However, it is not known whether anti-c-fms antibodies can inhibit osteoclast formation via cell–cell contact in co-cultures of osteoblasts and osteoclast precursors. 

In the present study, we show the inhibitory effect of anti-c-fms antibodies on osteoclast formation in in vitro co-culture and osteoclast formation and bone resorption in osteoporosis using OVX mice.

## 2. Results

### 2.1. Anti-c-fms Antibodies Inhibit Osteoclast Formation and Bone Resorption in an Osteoblast–Osteoclast Co-Culture System

**Figure 1:**
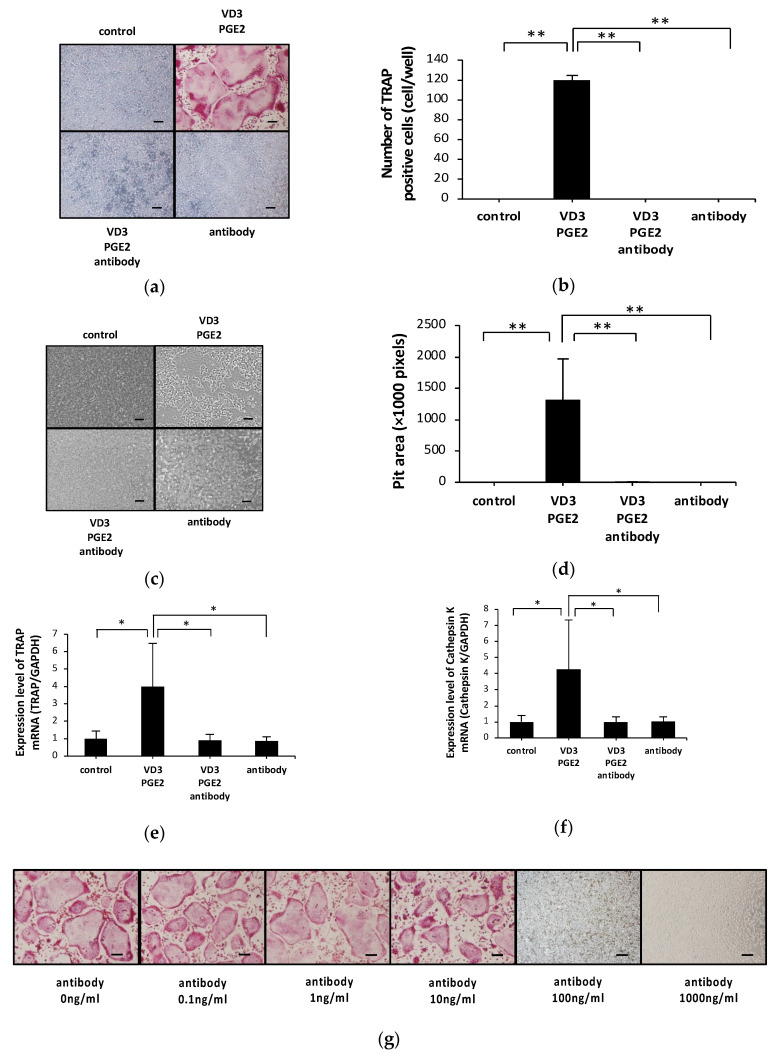

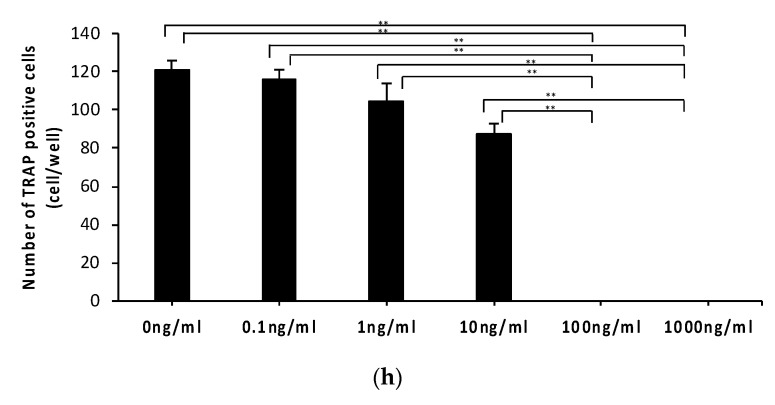
Anti-c-fms antibodies inhibit osteoclast formation and bone resorption in osteoblast–osteoclast precursor co-culture in vitro. (**a**) Microscopy images and (**b**) numbers of TRAP-positive cells in osteoblast–osteoclast precursor co-cultures treated with PBS, 1,25(OH)_2_D_3_ and PGE2, 1,25(OH)_2_D_3_ and PGE2 in the presence of anti-c-fms antibody (100 ng/mL), or anti-c-fms antibody (100 ng/mL) alone. (**c**) Microscopy images and (**d**) the percentage of resorption pits in osteoblast–osteoclast precursor co-culture treated with PBS, 1,25(OH)_2_D_3_ and PGE2, 1,25(OH)_2_D_3_ and PGE2 in the presence of anti-c-fms antibody (100 ng/mL), or anti-c-fms antibody (100 ng/mL) alone. (**e**) Levels of TRAP and (**f**) cathepsin K mRNA in osteoblast–osteoclast precursor co-cultures treated with PBS, 1,25(OH)_2_D_3_ and PGE2, 1,25(OH)_2_D_3_ and PGE2 in the presence of anti-c-fms antibody (100 ng/mL), or anti-c-fms antibody (100 ng/mL) alone analyzed by real-time PCR. mRNA levels for TRAP and cathepsin K were normalized to GAPDH. (**g**) Microscopy images and (**h**) numbers of TRAP-positive cells in osteoblast–osteoclast precursor co-cultures treated with 1,25(OH)_2_D_3_ and PGE2 in the presence of various anti-c-fms antibody doses. Results are expressed as means ± SD. Statistical significance of differences between groups were determined using Scheffe’s test (*n* = 4; ** *p* < 0.01 * *p* < 0.05). Scale bar = 200 μm.

We investigated whether anti-c-fms antibodies inhibit osteoclast formation in an osteoblast–osteoclast co-culture system. Treatment with 1,25-dihydroxyvitamin D3 [1,25(OH)_2_D_3_] and prostaglandin E2 (PGE2) induced many TRAP-positive cells in the co-culture system. However, addition of anti-c-fms antibodies (100 ng/mL) reduced the number of TRAP-positive cells (Figure 1a,b). In our previous study, 100 ng/mL anti-c-fms antibody inhibited both RANKL-induced osteoclast formation and TNF-α-induced osteoclast formation in vitro [26]. Therefore, we used 100 ng/mL anti-c-fms antibodies in this study. Anti-c-fms antibodies also inhibited the formation of resorption pits (Figure 1c,d). Real-time RT-PCR revealed that TRAP and cathepsin K mRNA levels were increased by 1,25(OH)_2_D_3_ and PGE2 treatment but that the addition of anti-c-fms antibodies inhibited TRAP and cathepsin K mRNA expression (Figure 1e,f). Osteoclast formation in the osteoblast–osteoclast cell co-culture system in response to several doses of anti-c-fms antibody is shown in Figure 1g,h. Anti-c-fms antibodies at 0.1, 1 and 10 ng/mL did not significantly reduce the number of osteoclasts compared with the control (0 ng/mL). However, there was a significant decrease in the number of osteoclasts with 100 and 1000 ng/mL anti-c-fms antibody compared with the control.

### 2.2. Anti-c-fms Antibodies Do Not Affect RANKL and OPG mRNA Levels or Osteoblast Viability or Differentiation

**Figure 2:**
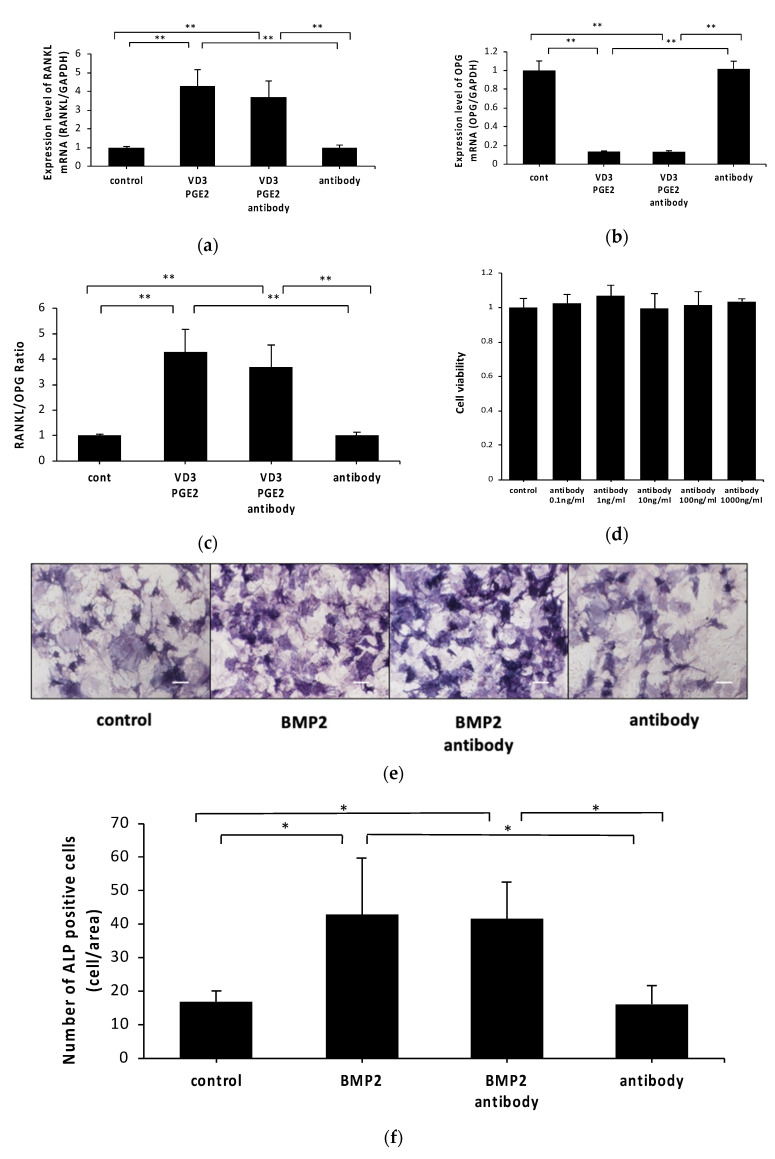
Anti-c-fms antibodies do not alter 1,25(OH)_2_D_3_ and PGE2-affected RANKL OPG mRNA levels, cell viability or osteogenic differentiation in osteoblasts. (**a**) Levels of RANKL and (**b**) OPG mRNA in osteoblasts treated with PBS, 1,25(OH)_2_D_3_ and PGE2, 1,25(OH)_2_D_3_ and PGE2 in the presence of anti-c-fms antibody (100 ng/mL), or anti-c-fms antibody (100 ng/mL) alone analyzed by real-time PCR. Levels of RANKL and M-CSF mRNA were normalized to those of glyceraldehyde 3-phosphate dehydrogenase (GAPDH). (**c**) OPG/RANKL ratio in osteoblasts. (**d**) Cell viability of osteoblasts treated with various doses of anti-c-fms antibody for 3 days. Cell viability was evaluated using cell counting kit-8. Data are presented as percentage activity relative to the activity in the culture without anti-c-fms antibody and are expressed as the mean ± SD. (**e**) Osteoblasts were seeded in 96-well plates at a density of 5 × 10^3^ cells/well in α-MEM containing BMP2 with anti-c-fms antibody (100 ng/mL) for 7 days. (**f**) Quantitative analysis of ALP-positive cells after treatment with anti-c-fms antibody (100 ng/mL) and BMP2. Data are presented as the number of ALP-positive cells in cultures with or without BMP2 and anti-c-fms antibody. Statistical significance of differences between groups was determined using Scheffe’s test (*n* = 4; ** *p* < 0.01 * *p* < 0.05). Scale bar = 200 μm.

We evaluated whether anti-c-fms antibodies inhibited vitamin D3 and PGE2-induced RANKL expression and 1,25(OH)_2_D_3_ and PGE2-inhibited OPG expression in osteoblasts in vitro. RANKL mRNA levels were higher in 1,25(OH)_2_D_3_ and PGE2-treated osteoblasts than in control cells and anti-c-fms antibody (100 ng/mL)-only-treated osteoblasts. OPG mRNA levels were lower in 1,25(OH)_2_D_3_ and PGE2-treated osteoblasts than in control and anti-c-fms antibody (100 ng/mL)-only-treated osteoblasts. However, osteoblasts treated with vitamin D3, PGE2 and anti-c-fms antibody (100 ng/mL) demonstrated similar RANKL and OPG mRNA levels to osteoblasts treated with 1,25(OH)_2_D_3_ and PGE2 alone (Figure 2a,b). The RANKL/OPG ratio was not different between antibody-treated osteoblasts and non-treated osteoblasts (Figure 2c). Osteoblasts cultured with anti-c-fms are shown in Figure 2d. Anti-c-fms antibodies at 1, 10, 100 and 1000 ng/mL did not significantly reduce the cell viability of osteoblasts compared with the control (0 ng/mL). These results show that the inhibitory effect of anti-c-fms antibodies on osteoclast formation in our co-culture system may not be related to the direct anti-c-fms antibody action on RANKL and OPG expression in osteoblasts.

We also evaluated the effect of anti-c-fms antibodies on osteogenic differentiation. To evaluate the effect of the anti-c-fms antibody on osteogenesis, osteoblasts were cultured in α-MEM complete medium with or without 100 ng/mL bone morphogenetic protein (BMP)2 and 100 ng/mL anti-c-fms antibody for seven days. We performed alkaline phosphatase (ALP) staining after seven days. ALP expression levels were higher in BMP2-treated osteoblasts than in control and anti-c-fms antibody-only-treated osteoblasts. However, osteoblasts treated with BMP2 and anti-c-fms antibodies demonstrated a similar number of ALP-positive cells compared with those treated with BMP2 alone (Figure 2e,f).

### 2.3. Anti-c-fms Antibodies Inhibit Bone Resorption and Bone Loss in OVX Mice

**Figure 3:**
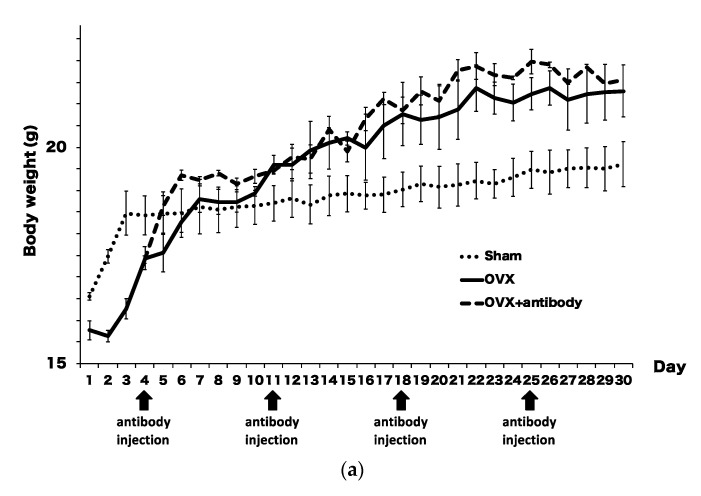

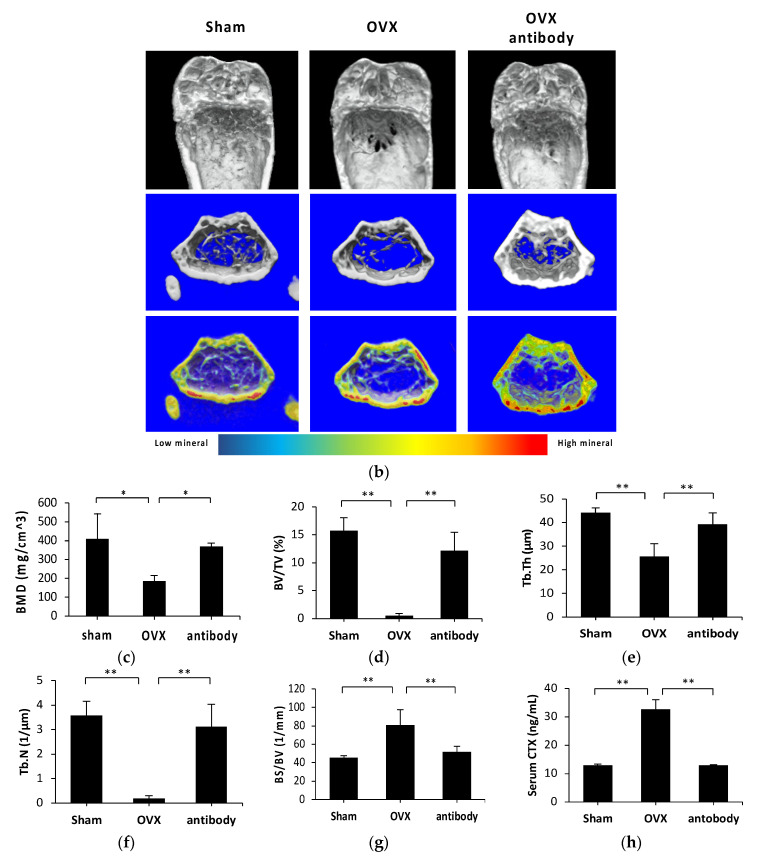
Anti-c-fms antibody inhibited bone resorption and bone loss in ovariectomized (OVX) mice. (**a**) Body weight changes in OVX mice treated with anti-c-fms antibody. OVX mice were treated with 500 μg/week/mouse anti-c-fms antibody (injection on day 4, day 11, day 18 and day 25 after operation). The date of injection of the antibody is indicated by the arrow. (**b**) Representative micro-CT images of trabecular bone microarchitecture in femurs. Thirty days from the start of the experiment, femurs from OVX mice, OVX mice treated with anti-c-fms antibody and sham mice were assessed by micro-CT. Phantoms with known BMD were imaged under the same conditions as the bones, and BMD images of cortical and trabecular bone were created from the CT images using the BMD values as pixel values in the third set of pictures. The height of the BMD is shown in color scale. Quantitative measurements of femur trabecular bone-related morphometric parameters, including (**c**) BMD, (**d**) BV/TV, (**e**) Tb.Th, (**f**) Tb.N and (**g**) BS/BV (*n* = 4). (**h**) Serum levels of CTX were assessed using a CTX Assay kit. Data are expressed as the mean ± standard deviation (SD). Statistical significance of differences between groups was determined using Scheffe’s test (*n* = 4; ** *p* < 0.01 * *p* < 0.05).

To confirm the success of ovariectomization, we measured the body weight of OVX mice every day. Significant increases in body weight were detected in OVX mice and OVX mice treated with anti-c-fms antibody compared with sham mice. There was no significant difference in the mean body weight between OVX mice and OVX mice treated with anti-c-fms antibody (500 μg/week/mouse). Average body weights of OVX, OVX mice treated with anti-c-fms antibody and sham mice are shown in Figure 3a. These results indicated that anti-c-fms antibodies did not affect body weight increases in OVX mice. 

Three-dimensional reconstruction of trabecular microarchitecture in femurs showed differences among the groups (Figure 3b). OVX resulted in bone deterioration, as indicated by reduced bone mineral density (BMD), bone volume per tissue volume (BV/TV), trabecular thickness (Tb.Th) and trabecular number (Tb.N) compared with the sham group. However, bone surface area per bone volume (BS/BV) was significantly increased in the OVX group compared with the sham group. Anti-c-fms antibody treatment in OVX mice significantly reduced bone deterioration, as measured by BMD, BV/TV, Tb.Th and Tb.N (Figure 3c–f). Anti-c-fms antibody treatment in OVX mice significantly decreased BS/BV (Figure 3g).

The serum level of the bone resorption marker, C-terminal cross-linked telopeptide of type I collagen (CTX), was assessed using a mouse CTX assay kit. The CTX level in serum was significantly increased in the OVX group compared with the sham group. However, the serum CTX level in OVX mice after anti-c-fms antibody treatment was lower than that of untreated OVX mice (Figure 3h).

### 2.4. Anti-c-fms Antibodies Inhibit Osteoclast Formation in OVX Mice

**Figure 4:**
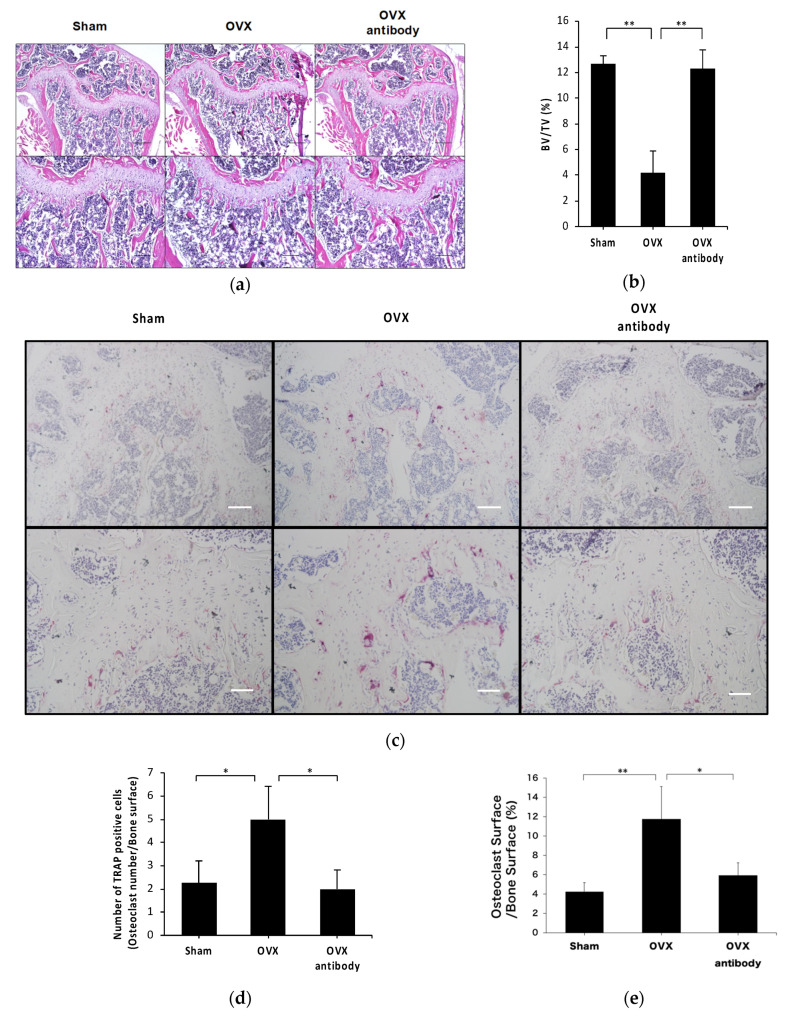
Anti-c-fms antibodies inhibited osteoclast formation in OVX mice. (**a**) Representative images of HE-stained distal femurs of OVX mice, OVX mice treated with anti-c-fms antibody and sham mice. Scale bar = 200 μm; scale bar = 100 μm in the enlarge pictures. Quantitative analysis of (**b**) BV/TV was performed using image J. (**c**) Representative images of TRAP-stained distal femurs in OVX mice, OVX mice treated with anti-c-fms antibody and sham mice. Scale bar = 100 μm; scale bar = 50 μm in the enlarged pictures. Quantitative analysis of (**d**) N.Oc/BS and (**e**) Oc.S/BS was performed using image J. Data are expressed as the mean ± standard deviation (SD). Statistical significance of differences between groups was determined using Scheffe’s test (*n* = 4; ** *p* < 0.01 * *p* < 0.05).

We evaluated the effect of anti-c-fms antibodies on bone resorption in OVX mice by histological assessment of hematoxylin and eosin (HE) staining. The BV/TV value in anti-c-fms antibody-injected OVX mice was higher than that in OVX mice (Figure 4a,b). Furthermore, we evaluated the effect of anti-c-fms antibodies on osteoclast formation in vivo by histological assessment of TRAP staining. The number of osteoclasts per unit bone surface area (N.Oc/BS) and unit osteoclast surface area per unit bone surface area (Oc.S/BS) were increased in OVX mice and anti-c-fms antibody treatment significantly decreased the N.Oc/BS and Oc.S/BS values. (Figure 4c–e).

## 3. Discussion

Osteoporosis is a systemic skeletal disease characterized by bone deterioration and decreased bone mass, leading to increased bone fragility and fracture rate [27]. The morphological characteristics of osteoporosis are bone resorption and decreased micro-architecture parameters, which result in reduced bone strength and increased risk of fractures [28]. In the present study, we showed the effect of anti-c-fms antibodies on osteoclast formation and bone resorption in osteoblast–osteoclast precursor co-culture and in OVX mice. We found that the anti-c-fms antibody inhibited osteoclast formation in osteoblast–osteoclast precursor co-culture in vitro. However, the anti-c-fms antibody did not change the RANKL or OPG expression during osteoblast differentiation in vitro. These results indicate that anti-c-fms antibodies directly affect osteoclast formation from osteoclast precursors in our co-culture system. Furthermore, we evaluated the effect of anti-c-fms antibodies on the OVX mouse model by micro-CT and histological analysis. Our results showed that anti-c-fms antibodies prevented OVX-associated bone loss and osteoclast formation in vivo. These results indicate the potential for therapeutic application of anti-c-fms antibodies for postmenopausal osteoporosis.

The effects of anti-c-fms antibodies on blocking RANKL-induced osteoclast formation and TNF-α-induced osteoclast formation have been studied in vitro by assessment of osteoclast precursors or bone marrow cells cultured with M-CSF and RANKL or TNF-α with or without anti-c-fms antibodies [13,17,26]. Anti-c-fms antibodies significantly inhibited osteoclast formation. These results indicated that the anti-c-fms antibody inhibited the effect of soluble M-CSF. It was recently reported that soluble RANKL deficiency does not affect the degree of bone loss, which suggests that membrane-bound RANKL via cell to cell contact plays an important role in osteoporosis pathology [25]. In in vitro co-culture of osteoblasts and osteoclast precursors, osteoclast formation requires signaling via soluble RANKL-mediated interactions or membrane-bound RANKL via cell–cell contact. In this study, we evaluated the effect of anti-c-fms antibodies on osteoclast formation in an osteoblast–osteoclast precursor co-culture and showed that the anti-c-fms antibody inhibited osteoclast formation. These results suggested that anti-c-fms antibodies inhibited osteoclast formation by inhibiting membrane-bound M-CSF via cell–cell contact. We also showed that anti-c-fms antibodies inhibited bone resorption in an osteoblast and osteoclast precursor co-culture. The results indicated that anti-c-fms antibodies also inhibited bone resorption by inhibiting membrane-bound M-CSF via cell–cell contact between osteoblast and osteoclast precursors. We also evaluated osteoclast formation in the osteoblast–osteoclast co-culture system using several anti-c-fms antibody doses and the number of osteoclasts formed was markedly decreased in a dose-dependent fashion. When 100 or 1000 ng/mL anti-c-fms antibody was used, there was a significant reduction in the number of osteoclasts compared with control cells. We previously showed that osteoclast formation in cultures treated with M-CSF and RANKL and with anti-c-fms antibody at 1 or 10 ng/mL did not significantly reduce the number of osteoclasts when compared with the 0 ng/mL control group; however, with anti-c-fms antibody at 100 or 1000 ng/mL, there was a significant reduction in the number of osteoclasts compared with other concentrations [26]. Furthermore, osteoclast formation in cultures treated with M-CSF and TNF-α and 1 ng/mL anti-c-fms antibody was similar to that with 0 ng/mL anti-c-fms antibody [26]. With 10, 100 or 1000 ng/mL anti-c-fms antibody, however, there was a significant reduction in the number of osteoclasts compared with other concentrations [26]. Moreover, osteoclast precursors cultured with M-CSF and anti-c-fms antibody at 0, 1, 10 or 100 ng/mL showed that there was no significant change in the number of osteoclast precursors among groups, but there was a significant decrease in the number of osteoclast precursors at 1000 ng/mL compared with other concentrations [26]. These results indicated that a concentration as high as 1000 ng/mL is necessary for inhibition of osteoclast precursor proliferation [26]. In this study, when we used 100 ng/mL anti-c-fms antibody, the number of osteoclasts was significantly decreased in osteoblast–osteoclast precursor co-cultures. These results showed that the amount of anti-c-fms antibody was similar to that needed in M-CSF and RANKL cultures to get the same effect.

The expression of RANKL is induced by 1,25(OH)_2_D_3_ and PGE2 [29,30]. In contrast, the expression of OPG is reduced in osteoblasts by 1,25(OH)_2_D_3_ [31]. In the present study, we evaluated the in vitro effects of anti-c-fms antibodies on 1,25(OH)_2_D_3_ and PGE2-induced RANKL and -decreased OPG expression in osteoblasts. RANKL mRNA levels were elevated in 1,25(OH)_2_D_3_ and PGE2-treated osteoblasts relative to control cells. OPG mRNA levels were decreased in 1,25(OH)_2_D_3_ and PGE2-treated osteoblasts relative to control cells. Furthermore, osteoblasts treated with 1,25(OH)_2_D_3_, PGE2 and anti-c-fms antibody exhibited similar levels of RANKL and OPG mRNA in osteoblasts that were treated with 1,25(OH)_2_D_3_ and PGE2. These results indicate that anti-c-fms antibodies may be unrelated to the increase in RANKL expression and decrease in OPG expression by vitamin D3 and PGE2 in osteoblasts. BMP2 is well known to induce osteoblastic differentiation of progenitor cells [32] and we evaluated in vitro effects of anti-c-fms antibodies on BMP2-induced osteoblast differentiation. ALP expression was elevated in BMP2-treated osteoblasts relative to control cells. Furthermore, osteoblasts treated with BMP2 and anti-c-fms antibody exhibited a similar ALP expression to that in osteoblasts treated with BMP2 only. These results indicated that anti-c-fms antibodies may be unrelated to BMP-induced osteoblastic differentiation.

OVX mice have been widely used as models of menopausal osteoporosis [33]. OVX causes an increase in body weight and a decrease in BMD and morphometric parameters [34]. Therefore, we chose OVX mice for a pre-clinical study to evaluate the effect of anti-c-fms antibodies on decreasing bone loss. The anti-c-fms antibody upregulated BMD and morphometric parameters indicating protection against bone loss. In a previous study, 500 μg anti-c-fms antibody injection was administrated every day for seven days to a mouse model of experimental arthritis. Osteoclast formation and bone resorption were inhibited in anti-c-fms antibody-injected arthritic mice [13]. Based on our previous study, in this study, we decided to administer 500 μg of anti-c-fms antibody, taking into consideration that the period of administration and the number of injections are different. We showed that anti-c-fms antibodies inhibited osteoclast formation and activated expression of RANKL and OPG in osteoblasts. Anti-c-fms antibodies inhibited osteoclast formation but did not affect osteoblast function. These data implicate that anti-c-fms antibodies may affect osteoclast precursors but not osteoblasts in OVX mice. However, in this study, we did not evaluate the effect of anti-c-fms antibody on osteoblast function in vivo. Further studies are needed to evaluate the effect of anti-c-fms antibody on osteoblast function in vivo. Anti-M-CSF antibodies prevent osteoclast formation and bone resorption in OVX mice [35], which suggests that M-CSF is a therapeutic target of bone loss in OVX mice. Recently, it has also been reported that anti-CSF-1R antibodies (2G2) inhibited bone loss in an osteoporosis mouse model [36]. Here, we used a different M-CSF blocking antibody, an anti-c-fms antibody (AFS98) and we obtained similar results for the inhibition of osteoporosis. These results provide strong evidence that blocking M-CSF inhibits osteoclast formation and bone resorption. M-CSF, a pleiotropic cytokine, is not only important for osteoclast differentiation but also proliferation and survival and is a regulator of key functions of monocytes and macrophages and their differentiation into mature phagocytes. Therefore, the role of M-CSF is not only bone regulation but also host defense [20,21]. In this study, we evaluated the effect of M-CSF by anti-c-fms antibody for osteoclast formation in OVX mice. However, further studies are needed to evaluate the effect of blocking M-CSF for other cells and functions such as host defense before clinical application.

In conclusion, our study suggests that anti-c-fms antibodies have the potential to be used as a therapeutic tool for postmenopausal osteoporosis.

## 4. Materials and Methods 

### 4.1. Animals

Twelve-week-old female C57BL6/J mice were obtained from CLEA Japan (Tokyo, Japan) and maintained in the animal facility of Tohoku University. We randomly assigned four mice to each experimental group. All animal husbandry and experimental procedures complied with the rules and regulations of Tohoku University. All experimental procedures conformed to Regulations for Animal Experiments and Related Activities at Tohoku University, and were reviewed by the Institutional Laboratory Animal Care and Use Committee of Tohoku University, and finally approved by the President of University (2019DnA-048–02, 4 July 2019).

AFS98 is a rat monoclonal anti-mouse c-fms antibody (IgG2a) that blocks M-CSF binding to its c-fms receptor and thus inhibits M-CSF-dependent colony formation and cell proliferation [37]. AFS98 hybridomas were maintained in HyQ-CCM1 medium (Hyclone, Logan, UT, USA). The antibodies were purified from culture medium using Protein G (Sigma Chemical Co., St. Louis, MO, USA) as previously reported [17].

### 4.2. Mouse Ovariectomy

Bilateral ovariectomy was performed on the OVX and the OVX with anti-c-fms groups, while a sham operation in which the ovaries were raised but not resected was performed for the sham group. Four days after ovariectomy, for the OVX with anti-c-fms group, 500 μg anti-c-fms was intraperitoneally injected every 7 days for 30 days (injection on day 4, 11, 18 and 25 after operation). The same volume of PBS was given to the OVX and sham groups. All mice were sacrificed at 30 days after surgery and femurs were obtained for micro-CT analysis and histological staining.

### 4.3. Preparation for Histological Evaluation

The femora were fixed in PBS-buffered 4% formaldehyde at 4°C for 5 days. The femora were then demineralized with 14% ethylenediaminetetraacetic acid (EDTA) at room temperature for 6 weeks, and then samples were processed using an automatic tissue-processor (Leica TP1020, Wetzlar, Germany). Femora were embedded in paraffin, sectioned at 5 μm using a microtome, and stained with HE. The analyzed area was the trabecular portion of the distal femur, distal to the epiphyseal plate. The regions of interest (ROI) of the trabecular bone extended 1.0 mm from the growth plate toward the diaphysis excluding the outer cortical bone. The area of the remaining cancellous bone in the ROI was measured and divided by the ROI, to give the BV/TV ratio. Each group consisted of four mice and four sections were quantified for each group. The paraffin sections were also stained with tartrate-resistant acid phosphatase (TRAP) solution, consisting of Fast Red Violet LB salt (Sigma Aldrich, St. Louis, MO, USA), naphthol AS-MX phosphate (Sigma Aldrich, St. Louis, MO, USA), 50 mM sodium tartrate and acetate buffer (pH 5.0), and were then counterstained with hematoxylin. TRAP-positive cells with three or more nuclei were considered as osteoclasts. Sample imaging was performed under a light microscope, and TRAP-positive cells were counted using ImageJ as previously reported [15].

### 4.4. Preparation of Osteoclast Precursors and Osteoblasts

Eight- to 10-week-old male C57BL6/J mice for preparation of osteoclast precursors were obtained from CLEA Japan (Tokyo, Japan). After euthanasia, the femora and tibiae of mice were aseptically removed and dissected free of adhering tissues. To obtain bone marrow cells, the bone ends were cut off using scissors and the marrow was flushed out using a 25-gauge needle and syringe prefilled withα-MEM (Sigma Aldrich, St. Louis, MO, USA). The cells were filtered through a 40-μm nylon cell strainer (Corning Inc., Corning, NY, USA). After washing with α-MEM, the cells were cultured in α-MEM containing 10% fetal bovine serum (FBS), penicillin G (100 IU/mL) and streptomycin (100 μg/mL) (Meiji Seika, Tokyo, Japan), and M-CSF for 3 days. Non-adherent cells were removed by rinsing twice with PBS. Then adherent cells were detached using a trypsin-EDTA solution (Sigma Aldrich, St. Louis, MO, USA). The cells were further cultured for 3 days with M-CSF. The adherent cells were used as osteoclast precursors [38]. Recombinant mouse M-CSF was made from an M-CSF expressing cell line [38].

Neonatal calvariae were dissected and serially digested in 0.2% collagenase solution (70 mM NaCl, 10 mM NaHCO_3,_ 60 mM sorbitol, 3 mM K_2_HPO_4_, 1 mM CaCl_2_, 0.1% BSA, 0.5% glucose and 25 mM HEPES) with agitation for 20 min at 37 °C and EDTA solution (5 mM EDTA in PBS containing 0.1% BSA; Wako, Japan) for 15 min. Digestions were performed as follows: fraction 1 (collagenase treatment), fraction 2 (EDTA treatment), fraction 3 (collagenase treatment), fraction 4 (collagenase treatment), and fraction 5 (EDTA treatment). The cells of fractions 3, 4, and 5 were collected and used in subsequent experiments. The harvested cells were cultured in α-MEM with 10% FBS for 3 days. Adherent cells were used as osteoblasts [39].

### 4.5. Co-Culture of Osteoclast Precursors and Osteoblasts

We constructed a primary osteoblast-osteoclast precursor co-culture system in the presence of 1,25(OH)_2_D_3_ and PGE2 with or without anti-c-fms antibody. Osteoclast precursors (5 × 10^4^ cells) and osteoblast cells (1 × 10^4^ cells) were cultured in 200 μL α-MEM with 10% FBS in 96-well plates in the presence of 10^−8^ M 1,25(OH)_2_D_3_ (Sigma Aldrich, St. Louis, MO, USA) and 10^−6^ M PGE2 (Sigma Aldrich, St. Louis, MO, USA) with and without anti-c-fms antibody (100 ng/mL). Medium was changed every other day. After 5 days, the cells were fixed in a 10% formalin solution for 30 min. Permeabilization of cells was performed with 0.2% Triton X-100 (Sigma Aldrich, St. Louis, MO, USA) for 5 min. Active osteoclasts were visualized using TRAP staining solution. TRAP-positive cells with three or more nuclei were considered as osteoclasts. The cells were counted under a light microscope. For resorption pit assays, osteoclast precursors (5 × 10^4^ cells) and osteoblastic cells (1 × 10^4^ cells) were cultured on Osteo Assay Plates 96 Well (Corning life Sciences, Corning, NY, USA) in the presence of 10^−8^ M 1,25(OH)_2_D_3_ and 10^−6^ M PGE2 with and without anti-c-fms antibody (100 ng/mL). After 5 days, formation of pits was evaluated. Data are expressed as resorption pits area per total surface area as described previously [40].

### 4.6. Preparation of RNA and Real-Time PCR Analysis

Total RNA was obtained using a RNeasy mini kit (Qiagen, Valencia, CA, USA). Osteoblasts or osteoblasts and osteoclast precursor co-cultures were incubated for 3 days in culture medium containing PBS, 1,25(OH)_2_D_3_ and PGE2, 1,25(OH)_2_D_3_, PGE2 and anti-c-fms antibody, or anti-c-fms antibody. Total RNA of osteoblast cultures and osteoblast and osteoclast precursor co-cultures was isolated using a RNeasy mini kit (Qiagen, Valencia, CA, USA). cDNA was synthesized using 2 μg total RNA with oligo-dT primers (Invitrogen, Carlsbad, CA, USA) and reverse transcriptase. The mRNA levels of cathepsin K and TRAP for co-cultured cells and RANKL and OPG for osteoblasts, relative to glyceraldehyde 3-phosphate dehydrogenase (*GAPDH*) mRNA, were assessed by real-time RT-PCR in a Thermal Cycler Dice Real Time system (Takara, Shiga, Japan). Each reaction consisted of 2 μL cDNA, a 23 μL mixture of SYBR Premix Ex Taq (Takara, Shiga, Japan), and 50 pmol/μL primers. PCR cycling conditions were as follows: initial denaturation stage (95 °C for 30 s), amplification stage (50 amplification cycles of a denaturation step of 95 °C for 5 s and an annealing step of 60 °C for 30 s), and a final dissociation stage (95 °C for 15 s, 60 °C for 30 s, and 95 °C for 15 s) as previously reported [40]. The primers used are listed in Table 1.

### 4.7. Evaluation of Bone Loss

Femora were fixed in 4% PBS-buffered formaldehyde for 5 days at 4 °C. Microfocus computed tomography (ScanXmate-E090; Comscan, Kanagawa, Japan) was used to scan the samples and reconstruction, which had a voltage of 90 kV, a current of 90 μA and a flat panel sensor readout of 1032 × 1012. The pixel size was 20 μm/pixel and a brass plate was used as a filter during scanning. It had a 360° rotation step and scanned at full 1 frame/second. ConeCTexpress (WhiteRabbit Corp., Tokyo, Japan) was used for reconstruction. TRI/3D-BON64 software R.7.00.06.0-H-64 (RATOC System Engineering, Tokyo, Japan) was used to make three-dimensional images of the femora. Quantitative morphometric assessments of bone characteristics were performed in an ROI delineated 1.0 mm below the growth plate. BS/BV, BV/TV, Tb.Th and Tb.N were evaluated as bone indexes.

### 4.8. Evaluation of Serum CTX Levels

Sera were obtained from the supernatant after centrifugation of blood samples from mice administrated with PBS, OVX or OVX with anti-c-fms antibody mice. Serum CTX levels were assessed using a mouse CTX assay kit (IDS, Boldon, UK). After the assay reaction, each well was evaluated by measuring absorbance at 450 nm with a microplate reader (Remote Sunrise; Tecan, Japan) with a reference wavelength of 620 nm as previously reported [41].

### 4.9. Evaluation of Cell Viability

Osteoblasts were seeded in a 96-well plate (1 × 10^4^ cells in 200 μL medium per well) in the presence of 10^−8^ M 1,25(OH)_2_D_3_ and 10^−6^ M PGE2 with or without different amounts of anti-c-fms antibody. Four replicates were assessed for each sample. After culture for 5 days, each well was washed with PBS and filled with 100 μL culture medium. Ten microliters of cell counting kit-8 (Dojin, Kumamoto, Japan) solution was immediately added to each well. The cells were incubated for 2 h at 37 °C. The absorbance at 450 nm was then measured for each well with a microplate reader (Remote Sunrise; Tecan, Japan) as previously reported [42].

### 4.10. Evaluation of Osteoblast Differentiation

Osteoblasts were cultured with PBS, BMP2, BMP2 and anti-c-fms antibody, or anti-c-fms antibody. After 7 days, ALP activity was assayed using an ALP stain kit (Wako, Tokyo, Japan) and the ALP-positive area evaluated.

### 4.11. Statistical Analysis

All data are expressed as the mean ± standard deviation. Differences between groups were assessed using Scheffe’s test. Statistical significance was assumed at a threshold of *p* < 0.05. Scheffe’s test is an ANOVA post hoc test. Data were analyzed using the computer software Excel statistics Statcel 3 (Microsoft, Seattle, WA, USA).

## Figures and Tables

**Table 1:** Primers used for real-time RT-PCR.

Gene	Sequence	
*GAPDH*	Forward	5′-GGTGGAGCCAAAAGGGTCA-3′
	Reverse	5′-GGGGGCTAAGCAGTTGGT-3′
*TRAP*	Forward	5′-AACTTGCGACCATTGTTA-3′
	Reverse	5′-GGGGACCTTTCGTTGATGT-3′
*RANKL*	Forward	5′-CCTGAGGCCAGCCATTT-3′
	Reverse	5′-CTTGGCCCAGCCTCGAT-3′
*OPG*	Forward	5′-ATCAGAGCCTCATCACCTT-3′
	Reverse	5′-TTAGGTCCAACTACAGAGGAAC-3′
*Cathepsin K*	Forward	5′-GCAGAGGTTGTACTATGA-3′
	Reverse	5′-GCAGGCGTTGTTCTTATT-3′

## Abbreviations

M-CSF	macrophage colony-stimulating factor
OVX	ovariectomy
RANKL	receptor activator of nuclear factor kb ligand
OPG	osteoprotegrin
TNF-α	Tumor necrosis factor-α
LPS	lipopolysaccharide
1,25(OH)_2_D_3_	1,25-dihydroxyvitamin D3
PGE2	prostaglandin E2
BMP	bone morphogenetic protein
ALP	alkaline phosphatase
BV/TV	bone volume per tissue volume
Tb.Th	trabecular thickness
Tb.N	trabecular number
BS/BV	bone surface area per bone volume
CTX	C-terminal cross-linked telopeptide of type I collagen
N.Oc/BS	number of osteoclast per bone surface
Oc.S/BS	osteoclast surface per bone surface
EDTA	ethylenediaminetetraacetic acid
